# Comparison of laparoscopic pyeloplasty vs. robot-assisted pyeloplasty for the management of ureteropelvic junction obstruction in children

**DOI:** 10.3389/fped.2022.1038454

**Published:** 2022-11-08

**Authors:** Marcos Pérez-Marchán, Marcos Pérez-Brayfield

**Affiliations:** ^1^ Department of Surgery at the University of Puerto Rico School of Medicine, Urology Section, San Juan, Puerto Rico; ^2^ Urology Section, Department of Surgery, School of Medicine, University of Puerto Rico, San Juan, Puerto Rico; ^3^ Section of Pediatric Urology, HIMA San Pablo Caguas Hospital, Caguas, Puerto Rico

**Keywords:** robotic, laparoscopy, pyeloplasty, pyeloplasty pediatrics, UPJ, UPJ obstruction

## Abstract

**Background:**

Ureteropelvic junction obstruction (UPJO) is a commonly observed abnormality in pediatric urology. Minimally invasive approaches have gained popularity in recent years. Studies have demonstrated excellent results with both laparoscopic pyeloplasty (LAP) and robot-assisted pyeloplasty (RAP). Few studies have compared the experience of both procedures performed in a single institution. Our objective is to compare laparoscopic pyeloplasty and robot-assisted pyeloplasty in the Puerto Rican pediatric population.

**Methods:**

We conducted a retrospective cohort study using our clinic's database on patients with UPJO that were operated by the same surgeon (MPB) from 2008 to 2019. Statistical analysis was conducted of demographics, preoperative studies, perioperative data and complications of both procedures. This study was approved by our local IRB committee.

**Results:**

A total of 86 patients that underwent pyeloplasty with at least 3 years of follow up were recorded for this study. Laparoscopic pyeloplasty and robot-assisted pyeloplasty were performed in 44 (51.1%) and 42 (48.8%) patients, respectively. Patient age ranged between 4 months and 17 years (LAP group - mean age of 6.19 years/RAP group - mean age of 7.07 years). Success rates was high in this series (LAP - 100% and RAP −95%). Using Wilcoxon signed rank test and Mann whitney sum test, significant differences between preoperative and postoperative hydronephrosis grading were observed in both LAP and RAP groups. However, no significant difference was seen regarding reduction of hydronephrosis grading when comparing both groups. No intraoperative complications were seen on either group.

**Conclusion:**

Both LAP and RAP are safe and effective procedures that can properly manage UPJO. Our study shows that, under experienced hands, pure laparoscopic pyeloplasty achieves comparable results to robotic assisted laparoscopic pyeloplasty. Pediatric urologists should be comfortable offering either of these approaches as they demonstrate high success rates in our pediatric population. Selection of LAP vs. RAP approach depends on the Surgeon's preference or experience, and on institutional availability. Minimally invasive therapies will continue to gain popularity with future advances in laparoscopic and robotic technology.

## Introduction

Ureteropelvic junction obstruction (UPJO) is commonly observed in the field of pediatric urology. Traditionally, the gold standard of surgical treatment for this disease was the open dismembered pyeloplasty which exhibited a success rate between 90% and 100%. The laparoscopic approach was adopted, yet it was accompanied with drawbacks including restrictive maneuvers and steep learning curve (1). Over the past decades, robotic surgery in pediatric urology has gained popularity (2). Since its implementation in 2002, annual increase rate of about 30% has been observed. More than 80% of minimally invasive pyeloplasty procedures, and 40% of pyeloplasty performed in children have been performed under this approach. Previous studies exist comparing Robot assisted pyeloplasty (RAP) with Laparoscopic pyeloplasty (LAP) when the two procedures were performed by different surgeons within the same institution. However, there are few studies that report single surgeons' experience with both LAP and RAP and compared their performance in the two procedures in a single institution (3). We aim to compare these techniques in the Puerto Rican pediatric population in our institution.

## Methods

We conducted a retrospective cohort study using our clinic's database on patients with UPJO that were operated by the same surgeon (MPB) from 2008 to 2019. Patients were deemed operable based on the following indications: Differential renal function under 40%, worsening hydronephrosis and/or flank pain. All patients had a renal scan and US performed preoperatively. Degree of hydronephrosis was assigned according to the Society of fetal urology hydronephrosis grading score ranging from grade 0 (no dilatation) to grade 4 (significant dilatation of renal pelvis and calyces, renal atrophy or cortical thinning). Our technique for both laparoscopic and robotic dismembered pyeloplasty are similar in nature. The only significant difference is the port size in which we use three 5 mm trocars in laparoscopy vs. three 8 mm trocars in Robotic surgery. Instruments used during the procedure include: 2 dissecting forceps, scissors, 2 needle drivers, and a suction device. The approach to the UPJ area can be transmesenteric for left sided UPJO or with medial mobilization of the colon for Right sided UPJO and with selected complicated left sided UPJO. Caution is needed during the initial dissection of the UPJ area to avoid injury to a lower pole crossing vessel. Tethered stitches using 3–0 prolene on a CT needle can be placed to the renal pelvis and proximal ureter to help with exposure and ease of the operation. All patients had an antegrade stent placed during the pyeloplasty (Both laparoscopic and robotic). All patients were followed with Renal Bladder Ultrasound (RBUS) 2 weeks postop and repeated every 4 months until resolution of hydronephrosis. Persistent or worsening hydronephrosis underwent postoperative studies. Success was defined as improvement of hydronephrosis, resolution of symptoms and no need for further surgical intervention. With this data, we analyzed demographics, preoperative studies, perioperative data and complications of both procedures. This study was approved by our local IRB committee.

## Results

A total of 86 patients that underwent pyeloplasty were recorded for this study. Patient age ranged between 4 months and 17 years (LAP group - mean age of 6.19 years/RAP group - mean age of 7.07 years). Laparoscopic pyeloplasty and robot-assisted pyeloplasty were performed in 44 (51.1%) and 42 (48.8%) patients, respectively. Laterality of the affected kidney was predominately on the left side in both groups (LAP group- left (26 patients), Right (18 patients); RAP group – Left (27 patients), Right (15 patients) (Table 1). Preoperative hydronephrosis was graded in both groups. Table 2 displays the following data: the LAP group has 24 patients with grade 4 hydronephrosis, 4 patients with grade 3 hydronephrosis and 2 patients with an unspecified grading. The RAP group had 30 patients with grade 4 hydronephrosis, 1 patient with grade 3 hydronephrosis and 4 patients with unspecified grading. Subsequently, postoperative hydronephrosis grading was also graded. The LAP group had 23 patients with grade 0 hydronephrosis, 5 patients with grade 2 hydronephrosis, and one patient with grade 3 hydronephrosis. The RAP group had 19 patients with grade 0 hydronephrosis, 13 patients with grade 2 hydronephrosis and 1 patient with grade 4 hydronephrosis. One patient in the RAP group with worsening hydronephrosis required a redo pyeloplasty which was accomplished *via* laparoscopy. The change in the selected procedure was due to patient's preference. The patient with residual SFU III hydronephrosis had a MAG 3 scan with Lasix performed which showed preservation of renal function and no evidence of obstruction. The operative length was an average of 100 min in laparoscopic pyeloplasty and 120 min in robot-assisted laparoscopic pyeloplasty. The average hospital stay for both procedures was 1 day. Success rates were high in this series (LAP - 100% and RAP −98%). Significant differences between preoperative and postoperative hydronephrosis grading were observed in both LAP and RAP groups. However, no significant difference was seen regarding reduction of hydronephrosis grading when comparing both groups. No complications were observed in either group. Although the exact learning curve for robotic pyeloplasty is unknown. We assume that the first 10 robotic cases were performed under our learning curve. The only patient with worsening hydronephrosis had a severe reaction around the UPJ area causing extrinsic compression; however, this was later corrected surgically. No significant differences were observed in preoperative hydronephrosis grading, laterality, operative time, hospital stay, or success rate (Table 3).

**Table 1:** Patient characteristics.

Type of procedure	Number of patients	Laterality	Left	Right
LAP	44		26	18
RAP	42		27	15
Total	86		53	33

Patient characteristics regarding surgical procedure and laterality of ureteropelvic junction obstruction.

**Table 2:** Hydronephrosis grading.

Preop hydronephrosis	Unspecified	0	II	III	IV	Total
RAP	4	N/a	N/a	1	30	35
LAP	2	N/a	N/a	4	24	30
Post op hydronephrosis						
RAP	N/a	19	13	0	1	33
LAP	N/a	23	5	1	0	29

Grading of hydronephrosis according to Society of fetal urology; grade 0 (no dilatation), grade 1 (dilatation of the renal pelvis without dilatation of the calyces, no parenchymal atrophy), grade 2 (dilatation of the renal pelvis and calyces, no parenchymal atrophy), grade 3 (moderate dilatation of the renal pelvis and calyces, blunting of fornices and flattening of papillae, mild cortical thinning), grade 4 (gross dilatation of the renal pelvis and calyces, renal atrophy or cortical thinning).

**Table 3:** Analysis.

Procedure type	RAP	LAP
Avg Op time (min)	120	100
Avg Hospital time (days)	1	1
Success rate (%)	98	100
Complications (# of pts)	1	0

Perioperative data comparing robot-assisted pyeloplasty vs. laparoscopic pyeloplasty.

A two-tailed Wilcoxon signed rank test was conducted to examine whether there was a significant difference between Preop hydro and Post hydro for laparoscopic pyeloplasty (LAP). The results of the two-tailed Wilcoxon signed rank test were significant based on an alpha value of.05, *V* = 406.00, *z* = −4.76, *p* < 0.001. The median of Preop hydro (*Mdn* = 4.00) was significantly larger than the median of Post hydro (*Mdn* = 0.00). The same test was conducted to examine whether there was a significant difference between Preop hydro and Post hydro for robotic assisted pyeloplasty (RAP). The results of the two-tailed Wilcoxon signed rank test were significant based on an alpha value of 0.05, *V* = 528.00, *z* = −5.05, *p* < 0.001. The median of Preop hydro (*Mdn* = 4.00) was significantly larger than the median of Post hydro (*Mdn* = 0.00). According to these results, the differences in pre and post-operative hydronephrosis grading were not due to random variation in either group (Tables 4–6). A two-tailed Mann-Whitney two-sample rank-sum test was conducted to examine whether there were significant differences in Reduction of Hydronephrosis (Pre to Post) between the types of Pyeloplasty. There were 28 observations in group LAP and 33 observations in group RAP which could be analyzed with this test. The result of the two-tailed Mann-Whitney *U* test was not significant based on an alpha value of.05, *U* = 557, *z* = −1.52, *p* = 0.130. The mean rank for group LAP was 34.39 and the mean rank for group RAP was 28.12 which suggests that the distribution of Reduction of hydronephrosis for group LAP (*Mdn* = 4.00) was not significantly different from the distribution of Reduction of hydronephrosis for the RAP (*Mdn* = 3.00) category. Table 7 presents the result of the two-tailed Mann-Whitney *U* test.

**Table 4:** Summary statistics table for interval and ratio variables by time_hydro_LAP*.*

Variable	*M*	*SD*	*n*	*SE_M_*	Min	Max	Skewness	Kurtosis	*Mdn*	Mode
Hydro_LAP										
PRE	3.86	0.36	28	0.07	3.00	4.00	−2.04	2.17	4.00	4.00
POST	0.47	0.90	30	0.16	0.00	3.00	1.55	0.81	0.00	0.00

“-” indicates the statistic is undefined due to constant data or an insufficient sample size.

Statistical analysis of hydronephrosis grading before and after laparoscopic pyeloplasty.

**Table 5:** Summary statistics table for interval and ratio variables by time_hydro_RAP.

Variable	*M*	*SD*	*n*	*SE_M_*	Min	Max	Skewness	Kurtosis	*Mdn*	Mode
Hydro_RAP										
PRE	3.88	0.42	33	0.07	2.00	4.00	−3.52	11.82	4.00	4.00
POST	0.91	1.11	34	0.19	0.00	4.00	0.71	−0.49	0.00	0.00

“-” indicates the statistic is undefined due to constant data or an insufficient sample size.

Statistical analysis of hydronephrosis grading before and after robot assisted pyeloplasty.

**Table 6:** Median hydronephrosis grade.

	SFU hydronephrosis grade		Z	*P*-Value*
	Median (Interquartile Range)			
Type of procedure				
	Pre	Post		
LAP	4.00 (4-4)	0.00 (0–0)	−4.76	<0.001
RAP	4.00 (4-4)	0.00 (0–2)	−5.05	<0.001

* Wilcoxon signed rank test.

Using median SFU hydronephrosis grade, Wilcoxon signed rank test was used to assess difference in pre and post procedural hydronephrosis score in RAP and LAP.

**Table 7:** Two-Tailed mann-whitney test for reduction of hydronephrosis by pyeloplasty*.*

	Median				
Variable	LAP	RAP	U	z	*p*
Reduction_Hydro Pre to Post	4.00	3.00	557.00	−1.52	0.130

Acknowledgment to Elvis Santiago Rodriguez, MS who conducted the statistical review of this manuscript.

## Discussion

Robotic assisted laparoscopic surgery (RALS) is an extension of pure laparoscopic surgery much in the same way that laparoscopic surgery is an extension of open surgery. Dissection techniques and surgical fundamentals are essentially the same, irrespective of the platform elected by the surgeon. Each approach has its particular learning curve which is significantly steeper curve for the pure laparoscopic approach. Chammas Jr, M. F. et al. reports that the learning curve for laparoscopic pyeloplasty is steep, with some authors suggesting that a minimum of 50 surgical procedures with a high degree of complexity, performed for 1 year, with at least 1 procedure per week, is necessary to master the skills for this procedure (5). In regard to robot- assisted laparoscopic pyeloplasty, Kassite, I. et al. reports more than 41 cases are needed to achieve mastery while sorensen, M.D. et al. observed that after 15 to 20 cases, the procedure had similar outcomes and surgical success than that of open pyeloplasty (6, 7). Many would argue one of the fundamental challenges when it comes to pure laparoscopic surgery is intra-corporeal laparoscopic suturing and complex dissection both facilitated by the robotic approach. Traditional laparoscopic instruments lack dexterity and the ability to articulate. In cases where extensive re-construction is required, such as for pyeloplasty, the surgeon must necessarily be abundantly comfortable with pure laparoscopic intra-corporeal suturing. Upon its introduction into the market what made RALS so attractive for surgeons was that it made intra-corporeal suturing more facile and shortened the learning curve for laparoscopic surgery. Studies have demonstrated that laparoscopic novices perform significantly better on the robotic platform rather than on standard laparoscopic techniques (8, 9, 10). This is true for all metrics including total task time, instrument path length, and smoothness of motion. Nonetheless, the clinical principle which dictates that surgical approach should be tailored to surgeon preference still holds true. We acknowledge that robotic platform should reduce the learning care for most surgeons. In our experience, we found that significant experience with laparoscopic surgery reduces the robotic learning case to 1 to 2 cases.

Compared to the open approach a laparoscopic approach to pyeloplasty, whether robot assisted or not, has been linked to reduced pain scores, improved cosmetic results, shorter hospitalization, and rapid convalescence (4). Few studies have compared their experience with both laparoscopic pyeloplasty vs. robotic pyeloplasty by a single surgeon. Tam et al. evaluated 37 patients undergoing LAP and 26 patients undergoing RAP. Their overall success rate was 91.9% and 96.2% with LP and RAP respectively (*p* > 0.5). No differences were seen in operative times nor complications rates between the groups. They noted that RALS was technically easier and that it may facilitate training in minimally invasive pyeloplasty (3). Esposito et al. showed no differences in success rates in 30 LAP and 37 RAP. They reported RAP to be technically easier (11). Wong et al. evaluated their experience with LAP vs. RAP in patients less than 12 month of age. The operated on 22 patients with LAP approach and 24 with RAP. They reported success rate was 91% with LAP and 96% with RAP. Again no difference were identified between LAP and RAP but the authors refers a faster recovery and a shorter learning curve with RAP (12). Similarly, in our study we found that robotic assisted pyeloplasty (RAP) and laparoscopic pyeloplasty (LAP) are comparable in operative time, length of stay, success rate, and post-operative hydronephrosis grading. Only one patient in the entire cohort required re-operation due to worsening hydronephrosis after RAP. This might be explained by the fact that this operation took place early in the robotic learning curve. This patient underwent re-do LAP with excellent results. No intra-operative complications were seen on either group.

Robotic assisted surgery for UPJO has been shown to be 2.7 times costlier when compared to other surgical approaches (13). Varda and colleagues evaluated the national trends of UPJO treatment modalities in children including analysis of the available data on cost. Of note, when comparing laparoscopic vs. robotic approaches there was an average increase in costs of over $3,000 (14). Operating room costs were by far the greatest contributor to costs, with robotic supplies being the largest contributor to the rising cost. High volumes of RALS may be required for institutions to profit from the procedures as total investment cost is divided between an increased number of procedures performed. An estimated three to five robotic cases per week are necessary to profit from robotic surgery, which is a clear limitation for pediatric centers no matter their size (15). Andolffi et al. performed a systematic database search which included 19 original articles and 5 meta-analyses. They found that robotic approach showed benefits in decrease operative times, complications rates but found conflicting results regarding platform and equipment cost. They concluded that there is a need for further cost -effectiveness analyses (16). Although not every institution's reality, cost and access of medical equipment must be considered, especially in a setting like ours where robotic surgery is not available in every hospital and not necessarily covered by all medical insurance companies. In Puerto Rico, our biggest hurdle to widely offered robotic procedures are issues with insurance coverage for robotic procedures. The government medical insurance and most private medical insurance companies do not cover robotic procedures and those that cover require a significant out of pocket deductible. Only changes in the current healthcare model will allow for robotic procedures to be performed more commonly in our country.

Limitations to our study include the retrospective nature of our data collection. MAG – 3 renal scans were not performed in all patients in the postoperative periods. The study was only performed in patients with worsening hydronephrosis and/or symptomatic patients. Our experience comes from a surgeon with extensive experience with LAP prior to starting the robotic program that could explain the comparable operative times and success rates. This study is different from other articles in that we describe a series of a single surgeon using the same technique for both laparoscopic and robotic pyeloplasty that allows the comparing of both groups without variations that are operator dependent. This allow us to conclude that both techniques have comparable success rates and should be in the armamentarium in the treatment of UPJ obstruction.

## Conclusion

Both LAP and RAP are safe and effective procedures that can properly manage UPJO. Our study shows that, under experienced hands, pure laparoscopic pyeloplasty achieves comparable results to robotic assisted laparoscopic pyeloplasty. Pediatric urologists should be comfortable offering either of these approaches as they demonstrate high success rates in our pediatric population. Selection of LAP vs. RAP approach depends on the Surgeon's preference or experience, and on institutional availability. Minimally invasive therapies will continue to gain popularity with future advances in laparoscopic and robotic technology.

## Data Availability

The raw data supporting the conclusions of this article will be made available by the authors, without undue reservation.

## References

[1] Howe A Kozel Z Palmer L. Robotic surgery in pediatric urology. Asian J Urol. (2017) 4:55–67. 10.1016/j.ajur.2016.06.002 29264208 PMC5730905

[2] Ekin RG Celik O Ilbey YO. An up-to-date overview of minimally invasive treatment methods in ureteropelvic junction obstruction. Cent European J Urol. (2015) 68:245–51. 10.5173/ceju.2015.543 26251754 PMC4526614

[3] Tam YH Pang KKY Wong YS Chan KW Lee KH. From laparoscopic pyeloplasty to robot-assisted laparoscopic pyeloplasty in primary and reoperative repairs for ureteropelvic junction obstruction in children. J Laparoendosc Adv Surg Tech A. (2018) 28(8):1012–8. 10.1089/lap.2017.0561 29641368

[4] Ravish I Nerli R Reddy M Amarkhed S. Laparoscopic pyeloplasty compared with open pyeloplasty in children. J Endourol. (2007) 21(8):897–902. 10.1089/end.2006.0411 17867949

[5] Chammas MF Jr Mitre AI Hubert N Egrot C Hubert J. Robotic laparoscopic pyeloplasty. JSLS. (2014) 18(1):110–5. 10.4293/108680813X1369342251983 24680152 PMC3939324

[6] Kassite I Braik K Villemagne T Lardy H Binet A. The Learning Curve Of Robot-Assisted Laparoscopic Pyeloplasty In Children: a Multioutcome Approach. J Pediatr Urol. (2018) 14(6):570.e1–570.e10. 10.1016/j.jpurol.2018.07.019 30177385

[7] Sorensen MD Delostrinos C Johnson MH Grady RW Lendvay TS. Comparison of the Learning Curve and Outcomes of Robotic Assisted Pediatric Pyeloplasty. J Urol. (2011) 185(6):2517–22. 10.1016/j.juro.2011.01.021 21555027

[8] Chandra V Nehra D Parent R Woo R Reyes R Hernandez T A comparison of laparoscopic and robotic assisted suturing performance by experts and novices. Surgery. (2010) 147(6):830–9. 10.1016/j.surg.2009.11.002 20045162

[9] Stefanidis D Wang F Korndorffer J Bruce Dunne J Scott D. Robotic assistance improves intracorporeal suturing performance and safety in the operating room while decreasing operator workload. Surg Endosc. (2010) 24:377–82. 10.1007/s00464-009-0578-0 19536599

[10] Sumi Y Dhumane P Komeda K Dallemagne B Kuroda D Marescaux J. Learning curves in expert and non-expert laparoscopic surgeons for robotic suturing with the da vinci surgical system. J Robot Surg. (2013) 7:29–34. 10.1007/s11701-012-0336-5 27000889

[11] Esposito C Masieri L Blanc T Musleh L Ballouhey Q Fourcade L Robot-assisted vs laparoscopic pyeloplasty in children with uretero-pelvic junction obstruction (UPJO): technical considerations and results. J Pediatr Urol. (2019) 15(6):667.e1–e8. 10.1016/j.jpurol.2019.09.018 31734119

[12] Wong Y Pang K Tam Y. Comparing robot-assisted laparoscopic pyeloplasty vs. Laparoscopic pyeloplasty in infants aged 12 months or less. Front Pediatr. (2021) 9:647139. 10.3389/fped.2021.647139 34195160 PMC8236621

[13] Link RE Bhayani SB Kavoussi LR. A prospective comparison of robotic and laparoscopic pyeloplasty. Ann Surg. (2006) 243:486–91. 10.1097/01.sla.0000205626.71982.32 16552199 PMC1448961

[14] Varda BK Johnson EK Clark C Chung BI Nelson CP Chang SL. National trends of perioperative outcomes and costs for open, laparoscopic and robotic pediatric pyeloplasty. J Urol. (2014) 191:1090–5. 10.1016/j.juro.2013.10.077 24513164 PMC4154938

[15] Palmer KJ Lowe GJ Coughlin GD Patil N Patel VR. Launching a successful robotic surgery program. J Endourol. (2008) 22:819–24. 10.1089/end.2007.9824 18419223

[16] Andolfi C Adamic B Oommen J Gundeti M. Robot-assisted laparoscopic pyeloplasty in infants and children: is it superior to conventional laparoscopy? World J Urol. (2019) 38(8):1827–33. 10.1007/s00345-019-02943-z 31506749

